# Alcohol use behaviors and risk of metabolic syndrome in South Korean middle-aged men

**DOI:** 10.1186/1471-2458-11-489

**Published:** 2011-06-22

**Authors:** Jinhee Kim, Su-Kyung Chu, Kyungjoo Kim, Ju-Ryoung Moon

**Affiliations:** 1 National Evidence-based Healthcare Collaborating Agency, Seoul, South Korea; 2 Department of Nursing, Eulji University, Seoul, South Korea; 3 National Strategic Coordinating Center for Clinical Research, Seoul, South Korea; 4 Cardiac & Vascular Center, Samsung Medical Center, Seoul, South Korea

## Abstract

**Background:**

It is thought that small volumes of alcohol may have positive effects on health. However, excessive drinking results in serious health problems. An accurate method to determine individual alcohol use behaviors are needed to assess objectively the extent to which drinking affects health. This study investigated the association between risk of metabolic syndrome (MetS) and alcohol use behaviors in middle-aged South Korean men using the Alcohol Use Disorders Identification Test.

**Methods:**

This study used data from the South Korea National Health and Nutrition Examination (KNHANES) IV (2008), which extracted the standard survey household by using the proportional systematic sampling method. Data of 714 participants from KNHANES IV, 2008 were analyzed using Surveyfreq and Surveylogistic regression to investigate the association between MetS and alcohol use behaviors in middle-aged South Korean men.

**Results:**

After adjustment for education, smoking, and physical activity, alcohol use behaviors were significantly associated with an increased risk of hypertension [odds ratio (OR) = 2.54, 95% confidence interval (CI) = 1.5-4.06 in the hazardous group; OR = 2.99, 95% CI = 1.84-4.92 in the problem group]; impaired fasting glucose (OR = 2.15, 95% CI = 1.16-3.99 in the hazardous group; OR = 2.48, 95% CI = 1.42-4.33 in the problem group); dyslipidemia (OR = 2.19, 95% CI = 1.38-3.47 in the problem group); abdominal obesity (OR = 1.93, 95% CI = 1.17-3.19 in the hazardous group; OR = 1.85, 95% CI = 1.17-2.92 in the problem group); and MetS (OR = 2.16, 95% CI = 1.24-3.77 in the hazardous group; OR = 2.54, 95% CI = 1.41-4.58 in problem group).

**Conclusions:**

This study found that excessive alcohol use behaviors increased the risk of hypertension, diabetes, dyslipidemia, abdominal obesity, and MetS. Considering the rising rate of alcohol consumption and heavy drinking at single sittings, a culture of less risky alcohol consumption must be established to promote health among middle-aged men.

## Background

Middle-aged men can experience a sense of crisis and anxiety along with a deterioration of their physical health. This deterioration may be the result of attempting to perform a number of demanding roles, which may include parenting dependent children, providing care to aging relatives, and paid work [1]. In South Korea, before the 1980s, it was not expected that men would have much to do with the day-to-day care of their families. However, as societal values have shifted to emphasize individual quality of life, South Korean men have come to recognize their roles within the family as well as in the workplace [2]. However, this shift may have also increased the psychological pressure and sense of conflict felt within the middle-aged group, causing more stress, drinking, and chronic fatigue [3].

Previously known as X-syndrome or insulin resistance syndrome, metabolic syndrome (MetS) is a pathological condition characterized by diabetes, impaired glucose tolerance, dyslipidemia, hypertension, and obesity, and it contributes to the risk of death caused by cardiovascular disease and diabetes. Thus, early detection and management of this pathological condition are needed [4-6]. The prevalence of MetS differs among studies, but is generally in the range 8-24% in men and 7-46% in women [7]; the prevalence in South Korea has been reported as 27.4% in men and 20.9% in women (24.1% in South Korean adults) [8].

It is thought that small volumes of alcohol may have positive effects on health [9]. However, excessive drinking results in serious health problems, including an increased risk of cardiovascular disease, cancer, liver cirrhosis, chronic pancreatitis, and violence [10-12]. Alcohol-related health problems are of particular concern in South Korea, where the easy availability of alcohol and a tolerant and widespread "drinking culture" facilitate excessive consumption [13].

A large body of research has consistently shown that the pathogenesis of MetS is the result of factors related to both lifestyle (e.g., smoking, physical activity, and diet) and heredity [14-20]. However, studies examining the contribution of alcohol consumption to MetS have reported conflicting results [21-24]. This inconsistency was assumed to be derived from unreliable estimates of alcohol consumption, based on self-reported frequency of drinking and average daily alcohol consumption [25].

An accurate method to determine individual alcohol use behaviors are needed to assess objectively the extent to which drinking affects health. In addition, rather than relying upon simple variables, such as average frequency and daily consumption of alcohol, it necessary to assess the individual's alcohol use behaviors, which include alcohol-related health problems and symptoms of alcohol dependence.

Our study investigated the association between the risk of MetS and alcohol use behaviors in middle-aged South Korean men using the Alcohol Use Disorders Identification Test (AUDIT), developed by the World Health Organization (WHO)[26].

## Methods

### Study design

This cross-sectional study investigated the association between the risk of MetS and alcohol use behaviors in 40-50-year-old South Korean men.

### Data source and ethical considerations

Data from the second year of the fourth South Korean National Health and Nutrition Examination Survey (KNHANES IV) were used to investigate the association between the risk of MetS and alcohol use behaviors. KNHANES is a national survey performed every three years, beginning in 1998; it replaces the Korean National Health and Health Behavior Survey that were carried out every 3 years from 1962 to 1983 and the Korean National Nutrition Survey that was carried out every year from 1969 to 1995. The purpose of KNHANES is to establish national health policy and to develop a knowledge base for the evaluation of health risk factors and health indicators. The fourth survey, which was initiated in 2007, was later converted into a year-round investigation to improve weaknesses in survey items and the quality of results gained. The first (2007) and second (2008) years of KNHANES IV completed.

The KNHANES IV data (2008) extracted the standard survey household by using a systematic sampling method that was proportional to the number of households after the first stratified sampling conducted considering the region, administration district, and type of residence (apartment or regular house). Although the stratification variables were not considered, sex, and age range of the population were considered as the domain variables. KNHANES IV (2008) consisted of a Health Interview, Health Behavior Surveys, Health Examination Study, and Nutrition Survey. More details on the research methodology used to gather and analyze the second-year KNHANES IV data (2008) are available from the Guidelines for Use of KNHANES IV Raw Data [27] and Final Report of KNHANES IV Sampling Frame [28]. Raw data were requested directly from the KNHANES research group.

The National Evidence-based Healthcare Collaborating Agency (NECA) ethics committee approved the study.

### Study population

This study examined survey responses from 1,146 middle-aged men (40-59 years of age) who completed the Health Interview, Health Behavior Surveys, and Health Examination Study for KNHANES IV. However, we excluded 432 men whose documentation showed missing values for important analytical variables, as well as those who had been diagnosed and treated for hypertension and diabetes, leaving a total of 714 participants in this study.

### Measures

#### General characteristics

The general individual characteristics examined in our study included age, co-habitation, education, monthly average household income, smoking, and physical activity. Middle-aged men were split into two categories (40-49 and 50-59 years). Co-habitation referred to whether the individual lived with a spouse. Educational attainment was categorized as follows: no schooling, elementary school, middle school, high school, and college or post-graduate. Household income was categorized into low (< 1,290 thousand won), middle (1,300-3,390 thousand won), and high (> 3,400 thousand won) income classes, which has been recommended by other agencies [29]; these figures are based on the 2008 national monthly average income of 3,370 thousand won for households with more than two people and a minimum monthly living expense of 1,270 thousand won for a family of four people [29,30]. Regarding smoking, participants were categorized into nonsmokers with no past smoking experience, ex-smokers, and current smokers [23]. Physical activity was assessed on a yes/no basis, where "exercise" meant moderate activity for more than 30 minutes on 5 days per week or intense activity for more than 20 minutes on 3 days per week [31], or walking more than 30 minutes a day for more than 5 days per week.

#### Alcohol use behaviors

Individual alcohol use behaviors were categorized according to AUDIT score. AUDIT is a simple tool, developed by the WHO, to identify alcohol use behaviors in a primary care setting. The main items on this instrument are recent alcohol use, alcohol dependence symptoms, and alcohol-related problems. The test is made up of 10 questions summing to scores of 0 to 40; that is, each question has a set of possible responses, and each response has a score ranging from 0 to 4. Scores in the range of 0-7 represented low-risk drinking, 8-15 represented a medium-level alcohol problem, and scores of ≥16 represented a high-level alcohol problem[26]. This study categorized alcohol use into normal use (0-7), hazardous use (8-15), and problem group (16-40).

#### MetS

Based on guidance given in the National Cholesterol Education Program Adult Treatment Panel [32] and the Western Pacific Region's Asian Pacific Guideline[33], we defined MetS as satisfying three or more of the following criteria: (i) presence of hypertension, as determined by systolic blood pressure ≥ 130 mmHg or diastolic blood pressure ≥ 85 mmHg; (ii) fasting plasma glucose ≥ 110 mg/dL; (iii) serum triglyceride levels ≥ 150 mg/dL; (iv) serum high density lipoprotein (HDL) < 40 mg/dL; or (v) waist circumference > 90 cm.

### Statistical analyses

Considering the fact that the KNHANES IV data (2008) were extracted by the stratified sampling method, analyses were done using a survey procedure. Surveyfreq was used to compare differences in general characteristics, each of the MetS components, and the prevalence and number of MetS components according to alcohol use behaviors. Survey logistic regression was used to estimate the magnitude of the association between alcohol use behaviors and risk of MetS and each of its components. Data were adjusted for education, smoking, and physical activity. To test for a linear dose-response relationship, we scored the categories of alcohol use behaviors and entered the score as a continuous term in the regression model. Differences were considered statistically significant at *P *< 0.05. All analyses were performed using SAS version 9.2 (SAS Institute, Inc., Cary, NC, USA).

## Results

When classified according to alcohol consumption group, the participants showed significant differences according to educational attainment, smoking, and physical activity. The highest level of educational attainment was high school for most men in the normal (40.0%), hazardous (42.8%), and problem (36.1%) groups, but significant differences existed among these three groups (*p *= 0.038). Significant differences also existed with respect to smoking habits, with current smokers forming the largest part of the normal group (42.1%) compared to ex-smokers in the hazardous (51.8%) and problem (60.8%) groups (*p *< 0.001). Most men within the normal (79.1%), hazardous (84.5%), and problem (72.6%) groups reported that they did not exercise regularly, with significant statistical differences among the three groups (*p *= 0.006; Table 1).

**Table 1:** Characteristics of study participants according to alcohol use behaviors groups

Variables	Normal*		Hazardous*		Problem*		*p*
	
	Unweighted (n = 219)	Weighted	Unweighted (n = 263)	Weighted	Unweighted (n = 232)	Weighted	
Age (yr)							.827
40-49	137 (62.6)	1,012,233 (67.4)	164 (62.4)	1,185,640 (64.6)	147 (63.4)	1,039,470 (66.8)	
50-59	82 (37.4)	488,847 (32.6)	99 (37.6)	649,358 (35.4)	85 (36.6)	516,329 (33.2)	
Cohabitation							.494
Yes	202 (92.2)	1,380,253 (92.0)	240 (91.3)	1,684,197 (91.8)	203 (87.5)	1,384,220 (89.0)	
No	17 (7.8)	120,827 (8.0)	23 (8.7)	150,801 (8.2)	29 (12.5)	171,578 (11.0)	
Education							.038
Illiterate	4 (4.8)	29,814 (2.0)	9 (3.4)	65,687 (3.6)	11 (4.7)	50,012 (3.2)	
≤Elementary school	11 (5.0)	51,261 (3.4)	29 (11.0)	191,982 (10.5)	30 (12.9)	188,519 (12.1)	
≤Middle school	34 (15.5)	230,116 (15.3)	45 (17.1)	310,905 (16.9)	37 (15.9)	237,626 (15.3)	
≤High school	89 (40.6)	600,422 (40.0)	109 (41.4)	785,424 (42.8)	84 (36.2)	562,377 (36.1)	
College≤	81 (37.0)	589,467 (39.3)	71 (27.0)	481,001 (26.2)	70 (30.2)	517,265 (33.2)	
Monthly average household income							.056
Low	14 (6.4)	85,661 (5.7)	26 (9.9)	176,229 (9.6)	25 (10.8)	186,706 (12.0)	
Middle	64 (29.2)	463,187 (30.9)	83 (31.6)	645,415 (35.2)	57 (24.6)	387,808 (24.9)	
High	141 (64.4)	952,232 (63.4)	154 (58.6)	1,013,355 (55.2)	150 (64.7)	981,284 (63.1)	
Smoking							<.001
Nonsmoker	42 (19.2)	289,656 (19.3)	25 (9.5)	146,021 (8.0)	18 (7.8)	111,054 (7.1)	
Ex-smoker	84 (38.4)	578,843 (38.6)	138 (52.5)	951,424 (51.8)	140 (60.3)	946,529 (60.8)	
Current smoker	93 (42.5)	632,581 (42.1)	100 (38.0)	737,553 (40.2)	74 (31.9)	498,216 (32.0)	
Physical activity							.006
Yes	46 (21.0)	314,106 (20.9)	43 (16.3)	283,828 (15.5)	61 (26.3)	425,747 (27.4)	
No	173 (79.0)	1,186,975 (79.1)	220 (83.7)	1,551,170 (84.5)	171 (73.7)	1,130,051 (72.6)	

* Number of subjects (%)

Categorization according to alcohol use behaviors also revealed significant differences in the components of MetS, with significant differences in blood pressure, fasting plasma glucose, triglyceride levels, and waist circumference among the groups. The proportion of hypertensive subjects differed significantly among the normal (19.7%), hazardous (39.7%), and problem (41.3%) groups (*p *< 0.001), as did fasting plasma glucose levels (normal, 9.1%; hazardous, 17.4%; problem, 19.8%; *p *= 0.011), triglyceride levels (normal, 37.1%; hazardous, 46.2%; problem, 56.5%; *p *= 0.002), and waist circumferences (normal, 19.1%; hazardous, 31.3%; problem, 29.0%; *p *= 0.016). Overall, fewer men in the normal group (19.0%) were diagnosed with MetS, as compared to the hazardous (19.0%) and problem (21.3%) groups (*p *= 0.009; Table 2).

**Table 2:** Prevalence of MetS components in middle-aged men according to alcohol use behaviors groups

Variables	Normal*		Hazardous*		Problem*		*p*
	
	Unweighted (n = 219)	Weighted	Unweighted (n = 263)	Weighted	Unweighted (n = 232)	Weighted	
SBP ≥130/DBP ≥85 mmHg							<0.001
No	177 (80.8)	1,198,534 (80.3)	158 (60.1)	1,198,534 (65.2)	136 (58.6)	907,023 (58.7)	
Yes	42 (19.2)	293,853 (19.7)	105 (39.9)	729,217 (39.7)	96 (41.4)	639,345 (41.3)	
FPG (mg/dl)							0.011
<110	197 (90.0)	1,355,976 (90.9)	220 (83.7)	1,518,379 (82.6)	185 (79.7)	1,240,904 (80.2)	
≥110	22 (10.0)	136,411 (9.1)	43 (16.3)	319,974 (17.4)	47 (20.3)	305,465 (19.8)	
TG (mg/dl)							0.002
<150	139 (63.5)	938,024 (62.9)	140 (53.2)	989,432 (53.8)	101 (43.5)	673,118 (43.5)	
≥150	80 (36.5)	554,362 (37.1)	123 (46.8)	848,921 (46.2)	131 (56.5)	873,250 (56.5)	
HDL cholesterol (mg/dl)							0.989
≥40	198 (90.4)	1,344,251 (90.1)	235 (89.4)	1,649,815 (89.7)	206 (88.8)	1,385,960 (89.6)	
<40	21 (9.6)	148,136 (9.9)	28 (10.6)	188,538 (10.3)	26 (11.2)	160,409 (10.4)	
Waist circumference (cm)							0.016
≤90	174 (79.5)	1,207,352 (80.9)	181 (68.8)	1,262,234 (68.7)	161 (69.4)	1,098,043 (71.0)	
>90	45 (20.5)	285,035 (19.1)	82 (31.2)	576,120 (31.3)	71 (30.6)	448,326 (29.0)	
MetS							0.009
No	197 (90.0)	1,342,841 (81.0)	211 (80.2)	1,488,788 (81.0)	181 (78.0)	1,217,004 (78.7)	
Yes	22 (10.0)	149,546 (19.0)	52 (19.8)	349,566 (19.0)	51 (22.0)	329,364 (21.3)	

* Number of subjects (%)

SBP = systolic blood pressure; DBP = diastolic blood pressure; FPG = fasting plasma glucose; TG = triglyceride; HDL = high-density lipoprotein

Significant statistical differences were also observed in the number of concurrent risk factors according to alcohol use behaviors (*p *< 0.001). Within the normal group, most men showed zero (39.7%) or one (36.3%) risk factor, followed by a much smaller proportion experiencing two (14.0%). This distribution shifted upward in the hazardous group, with 26.9% showing zero factors, 25.8% showing one, and 28.3% showing two. This was similar in the problem group, with 20.2% of men showing zero factors, 32.0% showing one, and 26.5% showing two (Table 3).

**Table 3:** Number of metabolic syndrome components in middle-age male according to alcohol use behavior groups

Number of risk factors	Normal*		Hazardous*		Problem*		*p*
	
	Unweighted (n = 219)	Weighted	Unweighted (n = 263)	Weighted	Unweighted (n = 232)	Weighted	
0	87 (39.7)	592,311 (39.7)	70 (26.6)	493,767 (26.9)	45 (19.4)	311,794 (20.2)	<.001
1	83 (37.9)	541,024 (36.3)	69 (26.2)	473,883 (25.8)	72 (31.0)	495,033 (32.0)	
2	30 (13.7)	209,506 (14.0)	72 (27.4)	521,138 (28.3)	64 (27.6)	410,176 (26.5)	
3	21 (9.6)	140,423 (9.4)	42 (16.0)	274,690 (14.9)	36 (15.5)	229,349 (14.8)	
4	1 (0.5)	9,123 (0.6)	3 (1.1)	51,836 (2.8)	12 (5.2)	76,715 (5.0)	
5	0 (0.0)	0 (0.0)	2 (0.8)	23,039 (1.3)	3 (1.3)	23,300 (1.5)	

* Number of subjects (%)

After adjustment for education, smoking, and physical activity, alcohol use behaviors were significantly associated with an increased risk of hypertension [odds ratio (OR) = 2.54, 95% confidence interval (CI) = 1.59-4.06 in the hazardous group; OR = 2.99, 95% CI = 1.84-4.92 in the problem group]; impaired fasting glucose (OR = 2.15, 95% CI = 1.16-3.99 in the hazardous group; OR = 2.48, 95% CI = 1.42-4.33 in the problem group); dyslipidemia (OR = 2.19, 95% CI = 1.38-3.47 in the problem group); abdominal obesity (OR = 1.93, 95% CI = 1.17-3.19 in the hazardous group; OR = 1.85, 95% CI = 1.17-2.92 in the problem group); and MetS (OR = 2.16, 95% CI = 1.24-3.77 in the hazardous group; OR = 2.54, 95% CI = 1.41-4.58 in problem group). Additionally, a dose-response relationship existed between alcohol use behaviors and blood pressure (*p *< 0.001), fasting plasma glucose (*p *= 0.001), triglyceride level (*p *= 0.001), waist circumference (*p *= 0.008), and MetS (*p *= 0.002; Table 4).

**Table 4:** Adjusted ORs* and 95% CI of components of metabolic syndrome and metabolic syndrome associated with alcohol use behavior against normal group

Variables	SBP^†^≥ 130/DBP^†^≥ 85	*p*	FPG^‡ ^≥ 110	*p*	TG^‡ ^≥ 150	*p*	HDL-C^‡ ^< 40	*p*	WC^§ ^> 90	*p*	Metabolic syndrome	*p*
												
	Adjusted OR (95% CI)		Adjusted OR (95% CI)		Adjusted OR (95% CI)		Adjusted OR (95% CI)		Adjusted OR (95% CI)		Adjusted OR (95% CI)	
Normal	1.00		1.00		1.00		1.00		1.00		1.00	
Hazardous	2.54 (1.59-4.06)	<.001	2.15 (1.16-3.99)	.015	1.43 (0.94-2.17)	.092	1.04 (0.52-2.07)	.917	1.93 (1.17-3.19)	.011	2.16 (1.24-3.77)	.007
Problem	2.99 (1.83-4.92)	<.001	2.48 (1.42-4.33)	.001	2.19 (1.38-3.47)	.001	1.07 (0.54-2.13)	.839	1.85 (1.17-2.92)	.009	2.54 (1.41-4.58)	.002
*P *for linear trend	<.001		.001		.001		.838		.008		.002	

SBP = systolic blood pressure; DBP = diastolic blood pressure; FPG = fasting plasma glucose; TG = triglyceride; HDL-C = high-density lipoprotein cholesterol; WC = waist circumference; OR = odds ratio; CI = confidence interval.

* ORs were adjusted for education, smoking, and physical activity.

^†^Unit: mmHg

^‡ ^Unit: mg/dL

^§ ^Unit: cm

## Discussion

Our study investigated the association between the risk of MetS and alcohol use behaviors in middle-aged South Korean men. In total, 69.3% of our study population fell within the hazardous and problem groups, which have been deemed injurious to health. Although South Korea's annual alcohol consumption is lower than average, reaching only 8.1 l per year per person, as compared to the OECD average of 9.5 l [34], South Korea is one of the most individually intensive drinking nations, with 63.4% of people consuming > 5 servings of alcohol in one sitting, as reported in the Global Status Report on Alcohol 2004 [35]. South Koreans prefer to drink soju (Korean distilled spirits), which contains 15-30% ethanol [36] and makes up 40.4% of domestic alcohol consumption. Freiberg et al. [24] and Baik and Shin [21] have reported that drinking liquor increases the incidence of MetS to a greater degree than drinking beer or wine. Considering these conditions, policy makers need to intervene on a national level with regard to managing alcohol consumption among middle-aged men.

The prevalence of MetS found in the present study was 19.1%, which is lower than reported for males in the US (24.0%) and South Korea (27.4%), but is in agreement with the prevalence of 8-24% reported in previous studies [7,8,37]. The result could be attributable to the fact that the subjects in the present study were limited to middle-aged men.

Our results showed a significant association between the risk of MetS and alcohol use behaviors after accounting for various confounders in middle-aged men. These results are consistent with those of previous studies showing that alcohol consumption exceeding a certain level increases MetS [25,38,39]. Corrao et al. [10] have reported through meta-analysis that drinking >25 g/day of alcohol harms health and Alkerwi et al. [40] have reported that favorable metabolic effects seem to be restricted to alcohol consumption of <40 g/day among men.

Our study found that alcohol use behaviors were associated with particular components of MetS, including blood pressure, fasting plasma glucose, triglyceride level, and waist circumference, after adjusting for education, smoking, and physical activity. This means that increased alcohol consumption brings with it an increase in the risk of hypertension, impaired fasting glucose levels, high triglycerides, and abdominal obesity.

Excessive consumption of alcohol can lead to hypertension [23,39]. Although alcohol decreases blood pressure transiently during the first 2 hours after consumption, it subsequently increases blood pressure considerably [41]. A study by McFadden et al. [41] using meta-analysis reported that daily alcohol intake increased systolic blood pressure by 2.7 mmHg and diastolic blood pressure by 1.4 mmHg. Yoon et al. [23] also reported that alcohol consumption ≥30 g/d increased blood pressure. Consistent with our results, Baliunas et al. [42] and Chrysohoou et al. [39] reported that moderate alcohol consumption decreases the risk of diabetes, whereas excessive alcohol consumption increases the risk. Baliunas et al [42] also reported that alcohol consumption ≥60 g/d increased this risk. In addition, higher than moderate alcohol consumption has been found to increase the density of triglycerides in the bloodstream [39]. Yoon et al. [23] also reported that alcohol consumption ≥30 g/d increased the density of triglycerides. However, we observed a non-significant change in the risk of low HDL cholesterol in the hazardous and problem groups, which conflicts with previous studies showing that the level of HDL cholesterol increases with both excessive and moderate quantities of alcohol consumption[23,43-45]. The results of this study are consistent with those of Fan et al. [25], who showed that daily alcohol consumption above US dietary guidelines increased the risk of low HDL-cholesterol, although their study also failed to achieve statistical significance on this parameter. Duncan et al. [46] have reported that moderate alcohol consumption decreases waist circumference, whereas excessive quantities modulate the concentration of sex hormones, causing abdominal obesity [25,36,46]. We likewise found that men in the hazardous and problem groups showed an increase in the risk of abdominal obesity, as compared to the normal group.

Strengths of this study included the use of a large pool of population-based data, and the use of established assessment methods such as AUDIT. Nevertheless, this study was limited in several dimensions. First, given the cross-sectional design, we could not draw any causal inference regarding the associations found. Second, we could not subdivide the alcohol use behaviors of the normal group, making it impossible to examine the association between light alcohol use and MetS and MetS components. The J-shaped association was not confirmed in the relationship between alcohol use and MetS. However, given that the purpose of this study was to investigate the association between alcohol use behaviors and the risk of MetS, the analytical model of our study was adequate. Third, the study results were acquired by analysis of data from the second year of KNHANES IV (2008) and could be generalized to the whole South Korean population. However, because data that were missing important analytical values were excluded from the present study, the present study encountered limitations resulting from the failure to secure a large enough sample as well as limitations in the generalizability of the study findings to all middle-aged South Korean men. Also, the exclusion of subjects with missing values could have introduced selection bias into the study. Therefore, careful interpretation of the present study results is required, especially considering that the study subjects with problematic alcohol use behaviors were found to comprise 69.3% of all subjects (hazardous alcohol use, 37.5%; problematic alcohol use, 31.8%). These results should be confirmed through future studies. Our study did not consider eating habits and family history, both of which are important risk factors for MetS [43,47]. Further studies are warranted to examine these factors.

## Conclusions

The present study investigated the relationship between alcohol use behaviors and the risk of MetS in middle-aged men in South Korea using the second-year data of KNHANES IV. In conclusion, our study found that excessive alcohol use behaviors increased the risk of hypertension, diabetes, dyslipidemia, abdominal obesity, and MetS after adjustment for education, smoking, and physical activity. Additionally, a dose-response relationship existed between alcohol use behaviors and blood pressure, fasting plasma glucose, triglyceride level, waist circumference, and MetS. Considering the rising rate of alcohol consumption and heavy drinking at single sittings, a culture of less risky alcohol consumption must be established to promote health among middle-aged men.

## Competing interests

The authors declare that they have no competing interests.

## Authors' contributions

SKC conceived of the study and participated in its design and coordination. JK participated in the design and coordination of the study and drafted the manuscript. KK participated in the study design and performed the statistical analysis. JRM helped to draft the manuscript. All authors read and approved the final manuscript.

## Pre-publication history

The pre-publication history for this paper can be accessed here:

http://www.biomedcentral.com/1471-2458/11/489/prepub
